# Technological interventions and advances in the diagnosis of intramammary infections in animals with emphasis on bovine population—a review

**DOI:** 10.1080/01652176.2019.1642546

**Published:** 2019-07-09

**Authors:** Sandip Chakraborty, Kuldeep Dhama, Ruchi Tiwari, Mohd. Iqbal Yatoo, Sandip Kumar Khurana, Rekha Khandia, Ashok Munjal, Palanivelu Munuswamy, M. Asok Kumar, Mithilesh Singh, Rajendra Singh, Vivek Kumar Gupta, Wanpen Chaicumpa

**Affiliations:** a Department of Veterinary Microbiology, College of Veterinary Sciences & Animal Husbandry, West Tripura, India;; b Division of Pathology, ICAR-Indian Veterinary Research Institute, Bareilly, India;; c Department of Veterinary Microbiology and Immunology, College of Veterinary Sciences, Deen Dayal Upadhayay Pashu Chikitsa Vigyan Vishwavidyalay Evum Go-Anusandhan Sansthan (DUVASU), Mathura, India;; d Sher-E-Kashmir University of Agricultural Sciences and Technology of Kashmir, Srinagar, India;; e Central Institute for Research on Buffaloes, Hisar, India;; f Department of Biochemistry and Genetics, Barkatullah University, Bhopal, India;; g Immunology Section, ICAR-Indian Veterinary Research Institute, Bareilly, India;; h Centre for Animal Disease Research and Diagnosis, ICAR-Indian Veterinary Research Institute, Bareilly, India;; i Center of Research Excellence on Therapeutic Proteins and Antibody Engineering, Department of Parasitology, Faculty of Medicine Siriraj Hospital, Mahidol University, Bangkok, Thailand

**Keywords:** Bovine, cow, mastitis, diagnosis, review

## Abstract

Mastitis, an inflammation of the udder, is a challenging problem in dairy animals accounting for high economic losses. Disease complexity, degree of economic losses and increasing importance of the dairy industries along with public health concerns envisages devising appropriate diagnostics of mastitis, which can offer rapid, accurate and confirmatory diagnosis. The various diagnostic tests of mastitis have been divided into general or phenotypic and specific or genotypic tests. General or phenotypic tests are those that identify general alterations, which are not specific to any pathogen. Genotypic tests are specific, hence confirmatory for diagnosis of mastitis and include specific culture, polymerase chain reaction (PCR) and its various versions (e.g. qRT-PCR), loop-mediated isothermal amplification, lateral flow assays, nucleotide sequencing, matrix-assisted laser desorption ionization time-of-flight mass spectrometry, and other molecular diagnostic methods. However, for highly specific and confirmatory diagnosis, pure cultures still provide raw materials for more sophisticated diagnostic technological interventions like PCR and nucleotide sequencing. Diagnostic ability of like infra-red thermography (IRT) has been shown to be similar to California mastitis test and also differentiates clinical mastitis from subclinical mastitis cases. As such, IRT can become a convenient and portable diagnostic tool. Of note, magnetic nanoparticles-based colorimetric biosensor assay was developed by using for instance proteolytic activity of plasmin or anti-*S. aureus* antibody. Last but not least, microRNAs have been suggested to be potential biomarkers for diagnosing bovine mastitis. This review summarizes the various diagnostic tests available for detection of mastitis including diagnosis through general and specific technological interventions and advances.

## 1. Introduction

Timely and accurate diagnosis of intramammary infections (IMI) in animals, especially affecting the dairy industry, is one of the most important aspects for mastitis prevention, treatment and control (Pyörälä 2009; Down et al. 2016). However, it has become a real challenge in the dairy farming with regards to difficulties being faced up in diagnosing mastitis (Krömker and Leimbach 2017; Rainard et al. 2018). This is aggravated by the rising ethical concerns for treating the affected animal properly as well as public health issues that may arise due to the inability to timely diagnose (e.g. subclinical mastitis) or posing up difficulties in treatment (e.g. chronic mastitis) of this disease appropriately (Groot and van’t Hooft 2016; Rainard et al. 2018). In addition to these, the improper use of antibiotics (Krömker and Leimbach 2017; Kumar and Gupta 2018) contributes toward concerns of emerging antibiotic resistance and predisposes to the menace of antibiotic residues in milk and meat products of affected animals (Oliver and Murinda 2012; Preethirani et al. 2015). The ongoing need for developing appropriate diagnostics for mastitis has gained immense importance in the recent past owing to the heavy economic losses from this disease condition (Shaheen et al. 2016) and that timely diagnosis of the IMI in animals has both prognostic as well as therapeutic monitoring roles (Alluwaimi 2004; Lakshmi 2016). There are quite many diagnostics evaluated and available at laboratory level, whereas field applicability renders many useless. Detailed description of these diagnostics will reveal the current status of mastitis detection and future possibilities for exploration besides the principle of operation and detection of biomarkers associated with alterations in milk.

Mastitis is a kind of IMI resulting in inflammation of udder parenchyma, physico-chemico-biological changes in milk and structural changes in the mammary tissue (Alnakip et al. 2014; Constable et al. 2017). Various indicators or biomarkers are released and/or affected due to the alterations in milk that can serve as diagnostic markers for mastitis (Lam et al. 2009; Abdelmegid et al. 2017; Hussein et al. 2018). These may include physical, chemical and/or biological alterations or markers, like electrical conductivity (Khatun et al. 2018), pH (Ondiek et al. 2018), biochemicals (e.g. metabolic substances) (Afaf et al. 2016; Qayyum et al. 2016), proteins (e.g. amyloid A) (Hussein et al. 2018), peptides (Mansor et al. 2013), enzymes [e.g. *N*-acetyl-β-d-glucosaminidase, alkaline phosphatase (ALP), lactate dehydrogenase (LDH)] (Pyörälä 2003; Duarte et al. 2015; Patil et al. 2015), lactose (Pyörälä 2003), somatic cell count (SCC) (Jadhav et al. 2018), microbial load (Vakkamäki et al. 2017; Mishra et al. 2018) and some novel biomarkers (Hussein et al. 2018; Zandkarimi et al. 2018). These markers are detected by various diagnostic methods, ranging from conventional observations, SCC, California mastitis test (CMT) (Kandeel et al. 2018a, 2018b; Rossi et al. 2018) to advanced polymerase chain reaction (PCR) (Mahmmod 2013), loop-mediated isothermal amplification (LAMP) (Bosward et al. 2016), lateral flow assays (Cornelissen et al. 2016), genomic (Wu et al. 2015), transcriptomic (Younis et al. 2016; Kosciuczuk et al. 2017), and proteomic (Zhao et al. 2015) analyses, as well as nano- and micro-fabrication of portable devices (Duarte et al. 2015; Ashraf and Imran 2018); out of which some are advanced diagnostics for mastitis, either used alone or in combination.

Initially, clinical observation, CMT, and white side test (WST) were the main field diagnostic tests, whereas SCC, culture and isolation were laboratory-based methods (Kandeel et al. 2018a, 2018b; Rossi et al. 2018). However, outcome and interpretation of these diagnostic tests was neither reliable nor specific or confirmatory (Cremonesi et al. 2018; Derakhshani et al. 2018). In recent times, molecular diagnostics (El-Sayed et al. 2017) including PCR (Lima et al. 2018), real-time (quantitative) reverse transcription PCR (qRT-PCR) (Behera et al. 2018), LAMP (Tie et al. 2012; Sheet et al. 2016), nucleotide sequencing (Oultram et al. 2017) and lateral flow assays (Cornelissen et al. 2016) are being applied for overcoming shortcomings of previous tests and for specific diagnosis of mastitis. However, accuracy, sensitivity and specificity remain the main concern for all such tests (Ashraf and Imran 2018; Rossi et al. 2018). Novel emerging diagnostic technologies including transcriptomic, genomic and proteomic approaches and nanotechnology-based method as well as microfabrication of portable usually digital devices possessing superior diagnostic features, have scoped for improving diagnosis of mastitis both at microbial and biomarker levels (Duarte et al. 2015; Ashraf and Imran 2018). These multi-omics approaches have promising roles in future (Zoldan et al. 2017).

The various diagnostic tests of mastitis have been divided into general or phenotypic and specific or genotypic tests. General or phenotypic tests are those that identify general alterations, which are not specific to any pathogen. Specific or genotypic tests are those that specifically detect biomarkers and/or genetic material of pathogens and are specific to pathogens (Ruegg 2009; Areo et al. 2017). Phenotypic tests detect alterations in physico-chemico-biological characteristics of milk such as electric conductivity, pH, biochemical changes, CMT, SCC, non-specific culture assessing total bacterial count, and proteomics (Schabauer et al. 2014; Cameron et al. 2017). Genotypic tests are specific, hence confirmatory for diagnosis of mastitis; and include specific culture, PCR and its various versions (e.g. qRT-PCR) (Behera et al. 2018), LAMP (Sheet et al. 2016), lateral flow assays (Cornelissen et al. 2016), nucleotide sequencing (Oultram et al. 2017), matrix-assisted laser desorption ionization time-of-flight mass spectrometry (MALDI-TOF MS) (Barreiro et al. 2017), and other molecular diagnostic methods (Perreten et al. 2013; Schabauer et al. 2014; El-Sayed et al. 2017; Lisowska-Lysiak et al. 2018).

Although many reviews on mastitis diagnostics are available general classification, principle description, comparative diagnostic feasibility and field applicability are yet to be explored fully. Thus, this review provides details on various aspects of mastitis diagnostics including conventional phenotypic and molecular genotypic-based techniques. Diagnostic technological interventions like SCC, CMT, automatic digital tests, infra-red thermography (IRT), sensor-based mastitis detection systems, advanced mastitis diagnostics, and proteomic approaches have been enlisted under general diagnostics, whereas details about specific culture, PCR and its versions, nucleotide sequencing/molecular typing methods, specific immunoassays, mastitis specific biomarkers are provided under specific diagnosis. Nevertheless, combination of diagnostic methods can be used for better accuracy. In future, focus should be on economical, convenient, field applicable and more reliable diagnostic tests for early, rapid, and accurate diagnosis of mastitis.

## 2. Diagnostics through technological interventions

Mastitis diagnostic tests should facilitate timely, accurate and/or confirmatory diagnosis of (subclinical) mastitis thereby enabling application of appropriate intervention. In either case, these tests are based on detection of some peculiarities of pathogens, alterations in characteristics of milk, body fluids or udder, or biomarkers/indicators that reflect mastitis (Nyman et al. 2016a, 2016b; Dervishi et al. 2017). For convenience, these have been reviewed under headings general mastitis indicators/markers and specific (subclinical) mastitis indicators/markers.

### 2.1. General mastitis indicators/markers

These are phenotypic mastitis diagnostic tests as they indicate the general changes that may be visible or non-visible and which are not specific to any pathogen but are diagnostic to (subclinical) mastitis. They include physico-chemico-biological diagnostics (pH, electric conductivity, enzymes, biochemical molecules, non-specific culture), SCC, CMT, digital mastitis detection tests, intramammary thermography, bio-sensors, or proteomic approaches (Schabauer et al. 2014; Nyman et al. 2016a; Kandeel et al. 2018c).

#### 2.1.1. Physiochemical diagnostics

These include numerous physical, biochemical and/or markers that are altered during (subclinical) mastitis, either in milk, blood or serum. The physical ones include general appearance of milk, electrical conductivity (Khatun et al. 2018), and pH (Ondiek et al. 2018) and the biochemicals include various metabolic substances such as lactose (Pyörälä 2003; Qayyum et al. 2016), proteins (e.g. amyloid A) (Hussein et al. 2018) , peptides (Mansor et al. 2013), and enzymes [e.g. *N*-acetyl-β-d-glucosaminidase (Kalmus et al. 2013), LDH (Afaf et al. 2016), ALP (Patil et al. 2015; Afaf et al. 2016), or milk arginase (Kandemir et al. 2013)]. Enzyme-based diagnostic tests are usually not reliable as these may vary in other diseases also. However, serum ALP and calcium levels were significantly decreased, whereas C-reactive protein (CRP) and phosphorus levels were increased in mastitis-affected cows. In addition, no significant change was noted in serum LDH, aspartate aminotransferase (AST), gamma-glutamyl transferase (GGT), albumin, sodium, potassium and chloride levels (Afaf et al. 2016). Detection of these markers has evolved from conventional spectrophotometry to various novel diagnostics, like immunoassays (Addis et al. 2016; Hussein et al. 2018). These have high specificity and sensitivity but less field applicability. Nowadays, advanced digital automatic diagnostics like Affimilk, Draminsiki milk detector, or Portascan are being developed and evaluated for mastitis diagnosis (Godden et al. 2017; Kandeel et al. 2018a, 2018b, 2018c). Although they are convenient their accuracy is limited.

#### 2.1.2. Somatic cell count (SCC)

SCC has been found to be an ideal method for subclinical mastitis diagnosis. It is convenient and reliable (Patil et al. 2015). Measurement of SCC directly has high accuracy but is costly and remains unavailable in many instances (Patil et al. 2015; Souza et al. 2016). Previously, manual SCC was considered as a laborious procedure, either of individual samples or a group of samples, while interpretation and accuracy were questionable. Nowadays with novel advanced diagnostics (DeLaval cell counter, Fossomatic cell counter, PortaCheck^®^, Somaticell^®^), SCC has become easy and accurate and large number of samples can be analyzed with ease and automaticity (Persson and Olofsson 2011; Ferronatto et al. 2018). For measurement of SCC in many samples together, cell counters with high capacity (based on the principle of flow cytometry), Fossomatic cell counter, is generally used. This method is reliable for measuring SCC (Lam et al. 2009; Kandeel et al. 2018a). Similarly, Somaticell^®^ has been found comparable to microscopic SCC and CMT (Ferronatto et al. 2018). Although universal standards are being followed for SCC as diagnostic method to clinical (above 5,000,000 cells) or subclinical (above 200,000 cells) mastitis, numerous factors can affect these values. Shook et al. (2017) revealed that the relationship of IMI with SCC was stable over time and very consistent over seasons, diverse production systems and animal factors. Bobbo et al. (2018) found that alternative SCC including somatic cell score (SCS), SCS_150, SCS_SD, test day (TD) SCC > 400,000 ml^−1^ in the lactation and the ratio of TD SCC > 400,000 ml^−1^ to the total number of TD in lactation exhibited additive genetic variation which could be exploited for breeding in Italian Holstein breed for improving resistance to mastitis. Nyman et al. (2018) found that non-*aureus* staphylococci (NAS) affects SCC and persistent IMI without affecting milk yield.

#### 2.1.3. California mastitis test

CMT is an easy, fast and cost-effective method for estimating SCC (Persson and Olofsson 2011). Surf field mastitis test is also used for screening of milk samples initially. However, due to incorrect execution, the usability of the test remains questionable (Lam et al. 2009). The use of CMT is not recommended for detection of mastitis before four days after calving. However, the test is of high value for monitoring success of therapy on the basis of estimation of SCC following treatment. Generally, CMT has been observed as less accurate (87.4–90.8%), less sensitive and specific than other tests like SCC which has shown a sensitivity of 94.9–99.5% and specificity of 48.1–87.1% (Rossi et al. 2018). Besides, CMT is a time-consuming process; thus, not suitable for large number of samples especially in large farms, and hence it has been replaced by novel sensitive and rapid diagnostics techniques, like digital tests or bio-sensors.

#### 2.1.4. Automatic digital diagnostics

Automatic mastitis detection systems are the novel diagnostic methods that are field applicable, easy and rapid (provide instant results) (Godden et al. 2017; Kandeel et al. 2018c). These include milk checker, Fossomatic meter, Dramiński mastitis detector/Wykrywacz mastitis detector, DeLaval cell counter, Afimilk mastitis detector, UdderCheck^®^ test and PortaSCC^®^ test (Godden et al. 2017; Steele et al. 2018). They are based on either detection of physico-chemical-biological alterations in milk or udder or estimation of biomarkers in body fluids (milk, serum) related to mastitis (Godden et al. 2017; Kandeel et al. 2018c).

The accuracy of PortaSCC^®^ as well as DeLaval cell counter has been found more suitable to estimate SCC (Barratt et al. 2003; Kawai et al. 2013). Jadhav et al. (2018) have delineated subclinical mastitis from normal udder on the basis of SCC; Holstein Friesian cows were tested for SCC estimation by digital PortaCheck. CMT and PCR were used to diagnose subclinical mastitis. They found SCC value of 310,000 cells ml^−1^ as threshold to delineate subclinical mastitis from normal udder.

Nowadays, in addition to microbial identification and microbial load, resistance to antibiotics by microbes needs due care. Hence, novel diagnostic methods are being designed that simultaneously determine these aspects. Systems of identification (automated), *viz*., VITEK identification cards, are available for this purpose providing results of identification of bacteria and antibiotic sensitivity (Funke and Kissling 2005; Crowley et al. 2012). The system has been proven to be rapid, reliable and accurate (Funke and Kissling 2005; Elbehiry et al. 2016; Kırkan et al. 2018). This system has been able to identify 84.09–100% of *S. aureus* correctly and its accuracy is considered to be 90% (Funke and Kissling 2005; Elbehiry et al. 2016).

#### 2.1.5. Infra-red thermography

Novel diagnostic techniques, like IRT (Polat et al. 2010; Sathiyabarathi et al. 2016a) have also found applications in mastitis diagnosis. This could be a very simple future prospect for diagnosis of subclinical mastitis in the field. Zaninelli et al. (2018) evaluated IRT for diagnosis of mastitis and could relate it well to SCC. With some refinement and improvement, the IRT could be very useful and convenient on-farm tool in future. IRT is a simple, effective, on-site and noninvasive type of diagnostic method that is based on heat or thermal difference of skin or udder surface; hence reflecting in the form of images, which are helpful in diagnosis of inflammation of udder (Sathiyabarathi et al. 2016a). For detection of subclinical mastitis in cows, diagnostic sensitivity and specificity of IRT have been found to be 95.6 and 93.6%, respectively, as compared to 88.9 and 98.9% for CMT (Polat et al. 2010). IRT could differentiate clinical mastitis from subclinical mastitis cases in bovines and ovines (Martins et al. 2013; Sathiyabarathi et al. 2016b). Higher temperature in clinical and lower in subclinical cases have been recorded by IRT in sheep (Martins et al. 2013) and cattle (Sathiyabarathi et al. 2016b). Thus, IRT is a sensitive, farmer friendly and non-toxic diagnostic procedure that helps in early detection of mastitis. The highly sensitive thermal camera of the IRT can detect even minor changes in surface temperature or inflammation of udder and with the mobile-based application, the IRT can become a convenient and portable diagnostic tool (Sinha et al. 2018).

#### 2.1.6. Sensor-based mastitis detection systems

These diagnostic systems help in sensing the mastitis usually with minimal stress on the animal. These diagnostic systems are especially helpful in large farms. In organized large dairy farms, automatic milking or machine milking has replaced the manual milking. Identification of mastitis cases from such large number of animals needs automatic detection techniques by using appropriate sensing technology, such as in-line monitoring of somatic cell count (ISCC) sensing technique along with quarter-based electrical conductivity (EC) of milk, so that clinical mastitis cases can be recognized well in time. Based on this concept, a study was performed over 200 cows in New Zealand during 2006–2007 and the findings have led to recommendation on a combined use of ISCC and EC for diagnosis of clinical mastitis (Kamphuis et al. 2008). Likewise, many other sensor-based mastitis detection systems were used and their efficacies were compared by researchers. It is expected that such techniques should have minimum sensitivity of 80% and specificity of at least 90% and should have detection time window of 48 h. However, comparative results showed that wide variation exists in between different sensor-based models (Hogeveen et al. 2010).

Magnetic nanoparticles-based colorimetric biosensor assay was developed by using proteolytic activity of plasmin as biomarker because in cases of mastitis, plasmin enhances the proteolysis of casein and reduces the quality of milk. This assay can differentiate between the milk of healthy and mastitis-affected animals. Plasmin attached to magnetic nanoparticles was present as monolayer over the gold sensor surface and the enhanced golden color of the surface of the biosensor is a direct indication of plasmin proteolytic activity. This biosensor is very sensitive to detect small amounts (1 ng ml^−1^) of plasmin present *in vitro* in the milk samples (Chinnappan et al. 2017). In an experimental study conducted over 47 cows suspected for subclinical mastitis from 12 Portuguese dairies, magnetic nanoparticles were used for specific detection of both *Staphylococcus aureus* and other staphylococci. For immunology-based magnetic detection, anti *S. aureus* antibody and anti-*Staphylococcus* spp. antibody were used and the sensitivity was 57.1 and 79.3% and specificity was 75 and 50%, respectively, for these antibodies. It is obvious that these magnetic detection methods need further improvements (Duarte et al. 2017).

#### 2.1.7. Proteomic approach

Proteomic approach has not only been a promising field but also has proven quite beneficial in diagnosing, forecasting and preventing mastitis in dairy animals along with standardizing quality (Abdelmegid et al. 2017; Ryskaliyeva et al. 2018). This method facilitates identification of biomarkers of IMI besides confirming many similar approaches used previously in mastitis assessment (Abdelmegid et al. 2017).

For studying the dynamics of differential expression of proteins during bovine mastitis, researchers have taken good measures in spite of shortcomings like complexity of the sample. Among various kinds of mastitis in cattle, in subclinical mastitis bacterial culture is necessary to perform along with SCC. To enhance the sensitivity and specificity in diagnosis, milk proteins acting as putative biomarkers in case of subclinical mastitis caused by *S. aureus* are identified through proteomics. Studies based on proteomics and MALDI-TOF revealed that whey proteins such as β-1,4 galactosyltransferase, β_2_-microglobulin, complement 3, α1-acid glycoprotein, β-lactoglobulin A, α-S1 casein precursor and β-casein B obtained from healthy cows are different from the cows with subclinical mastitis. It showed that protein markers present in bovine whey during subclinical stage of mastitis could be used as diagnostic candidate by means of comparative proteomics (Bian et al. 2014).

Researchers combined the label-free quantitative proteomics and bioinformatics approaches for the detection of important proteins present in milk and whey involved in host defense during IMI by mass spectrometry (Abdelmegid et al. 2017). Advances have been made significantly in the 21st century for the identification of low abundance proteins in several fractions of cow milk during both pre-clinical and clinical stages (Boehmer et al. 2008, 2010a, 2010b; Danielsen et al. 2010). Various strategies such as MALDI-TOF MS following two-dimensional gel electrophoresis (2D-GE), combined uses of liquid chromatography and tandem mass spectrometry have been adopted for proteomic analyses of milk collected during bovine mastitis. Magro et al. (2018) proposed the analysis of hard protein corona on nanomaterials as a potential future perspective for developing rapid analytical protocols for detection and diagnosis of mastitis in cows. Proteomics has been applied for evaluation of modulation of the proteins in milk collected either from natural cases of mastitis or from milk of cows experimentally challenged with lipopolysaccharide (LPS) or infected with *E. coli* (Danielsen et al. 2010; Boehmer 2011).

Proteomic approaches have also been undertaken for investigation of proteolysis in bovine milk following infusion with lipoteichoic acid of *S. aureus* origin. Likewise, for the assessment of the modulation in cow milk proteomes, several strategies of quantification such as spectral counting, densitometry, and stable isotopes incorporation have been adopted. Overall, thorough identification of approximately 80 proteins in relation to the response of the host to IMI has been undertaken (Larsen et al. 2010; Boehmer 2011; Hettinga et al. 2011). Multilocus sequence typing (MLST), microarray, 2D-GE and proteomic studies were used to explore the genes and proteins responsible for virulence (capsular polysaccharides, exotoxins) and resistance in *S. aureus* for genetic and epidemiological analyses. These techniques revealed variation in the gene expression and resultant heterogeneity presented in protein profiles of bovine and human origins of *S. aureus* strains. Commonly expressed proteins were Atl, Aur, GlpQ, Hla, LtaS, Nuc, PdhB, SAB0846, SAB2176, SAB0566, SspA, and SspB as part of exoproteomes, while serine proteases SplB, C, F, superantigens SEC-bov, SEL and toxic shock syndrome toxin-1 (TSST-1) were variably and rarely expressed. Among all these proteins, SAB0846 was more commonly present in bovine strains than the human strains of *S. aureus* (Wolf et al. 2011).

Several proteins including pyruvate dehydrogenase, aureolysin, alkyltransferase-like protein, stringent starvation proteins A and B, etc. have been identified by proteomic comparison of divergent strains of *S. aureus* isolated from bovine mastitis samples. Proteins that exhibit variable patterns of expression include TSST-1 superantigen, penicillin binding protein 2 (PBP-2), hyaluronate lyase precursor A1 and A2, etc. Generation of data through comparative proteomic analyses has strongly supported the theory of immunomodulation by superantigen and its role in the pathogenesis of mastitis. Such analytical approach will further help in the generation of vaccines against *S. aureus* through characterization of targets as well as host specificity (Wolf et al. 2011). Study has been conducted to highlight the proteomic changes in *E. coli* also isolated either from fresh bovine milk or in laboratory media. It has been found that various proteins of *E. coli*, *viz*., siderophores, β-galactosidase, and LuxS (enzyme involved in production of hormone-like substance in the bacteria) have been either upregulated or highly expressed. Conversely, there was downregulation of flagellin and other flagellar proteins (Lippolis et al. 2009). Kusebauch et al. (2018) used selected reaction monitoring (SRM) mass spectrometry to quantify 13 host response proteins in milk. Here, *in vivo* challenge with products from Gram-negative bacteria (LPS from *E. coli*) and Gram-positive bacteria [peptidoglycan (PGN) from *S. aureus*] resulted in consistent upregulation of innate immune response proteins. LPS challenge caused more intense and faster immune response in comparison to PGN challenge. Many proteins are being explored for various diagnostic feasibilities. Lactoferrin found in bovine milk can be used as putative biomarkers for ELISA and proteomics-based diagnosis of disease in cattle (Van Altena et al. 2016).

### 2.2. Specific mastitis diagnostic tests

These include the genotypic type of mastitis diagnostic tests that specifically detect the pathogens that cause the subclinical mastitis or their genetic materials or to estimate the biomarkers that specifically relates to the pathogens or denote the alterations which are specific to the pathogen (Nyman et al. 2016b; Ashraf and Imran 2018). Hence, the tests are diagnosis specific of mastitis and are quantifiable. They include specific cultures, PCR or PCR/DNA-based molecular techniques, nucleotide sequencing or genomic approaches, MALDI-TOF, specific immunoassays and specific biomarkers.

#### 2.2.1. Specific culture

It is important to have the proper knowledge for the microbial/bacteriological etiology of (subclinical) mastitis, for which microbiological/bacteriological culture is utmost required (Abdelmegid et al. 2017). However, no growth is observed upon bacteriological examination of milk samples in 10–40% of cases in clinical mastitis at the quarter level. Various reasons may be responsible for such situation, *viz*., presence of very few organisms, or samples may contain pathogens, like *Mycoplasma* spp., which require special technique and media for culturing (Fox 2012) or the cultural conditions may not be feasible. Besides, presence of antibiotics in milk may inhibit microbial growth. Other factors include requirement of specific media for growth, presence of inhibitory substances in milk, and/or viable cells damaged lethally due to extreme handling. Crucial roles may be played by latent infections or shedding cycles in case of subclinical mastitis (Sears et al. 1990; Gundelach et al. 2011). Specific culture is essential for specific diagnosis. The rate of isolation of pathogens has increased gradually by using various testing methods such as specific culture media, incubation, freezing and increase in volume of inoculation (Lam et al. 2009).

From the cultural practices of using conventional media (e.g. nutrient agar or broth) for common bacteria like streptococci and staphylococci, and slightly specific media (e.g. MacConkey agar for Gram-negative bacteria) were evolved initially. This progressed to highly specific media, like Mannitol salt agar for *Staphylococcus* spp., Eosin Methylene Blue agar for *E. coli*, and pleuropneumonia-like organism (PPLO) medium for *Mycoplasma* spp. In case of clinical mastitis, it is pertinent from the diagnostic point of view to have the results of bacteriological culture at the earliest time available for optimizing the results for treatment. This reduces the cost of treatment and helps proper use of antibiotics effectively. This is the reason for development of various culturing systems commercially like Minnesota Easy Culture System II as well as Petrifilm system. Both these have proven to be user-friendly. In comparison to traditional methods of culture, these have good test properties, which are proven to be sufficient as effective tools. However, it is required to have greater experience and capability to read and interpret the results when Petrifilm system is used in comparison to the Minnesota system (Godden et al. 2007; Lam et al. 2009; Royster et al. 2014). For Minnesota Easy Culture System II Bi-Plate and Tri-Plate systems for identification of common mastitis pathogens in milk specificity and accuracy was high (>80%), and sensitivity was intermediate (>60%) to high (>80%) (Royster et al. 2014). Petrifilm system has a sensitivity of 93.8% and a specificity of 70.1% (McCarron et al. 2009). Ferreira et al. (2018) compared four commercial on-farm culture systems including Accumast, Minnesota Easy System, Staph, Strep and Gram-Negative (SSGN) and Staph, Strep and Gram-Negative chromogenic (SSGNC) Quad plates. Accumast was found to be the most accurate for detection of mastitic pathogens. Keller and Sundrum (2018) opined that the antimicrobial therapy or alternative remedies like homeopathy should be based on bacterial culture of suspected mastitic milk sample. Further, for highly specific and confirmatory diagnosis, pure cultures still provide raw materials for more sophisticated diagnostic technological interventions like PCR and nucleotide sequencing.

#### 2.2.2. PCR and its versions

The use of various molecular methods to detect pathogens have been increased since last two decades and in context of detection of variety of mastitis causing pathogens, description about the techniques based on PCR are available in the literature (Lakshmi 2016). PCR has been proven as a rapid (1–2 days period), sensitive (76.9–100%) and specific (63.3–98.7%) method for diagnosis of mastitis (Spittel and Hoedemaker 2012; Parker et al. 2017; Vidic et al. 2018). Bacterial culture methods are also employed for detection of mastitis pathogens, but are far less sensitive (32.2%) than PCR assays (70.6%) (Spittel and Hoedemaker 2012; Parker et al. 2017; Vidic et al. 2018). However, when bacterial culture is lacking, as in slow growing microbes, e.g. *Mycoplasma*, interpretation of the results of PCR is relatively difficult as there are chances of DNA contamination from other sources or species (Riffon et al. 2001; Gillespie and Oliver 2005; Keane et al. 2013). Studies advocated that molecular techniques are more sensitive and faster than the traditional laboratory culture techniques in detecting pathogen within less span of time and therefore are more helpful for the clinician to plan the treatment regimen early (Cantekin et al. 2015). PCR has been used for the identification of microbes causing subclinical mastitis too (El-Sayed et al. 2017). It is also being utilised for molecular detection of different organisms like *Staphylococcus* spp., *E. coli* and *Mycoplasma* in milk samples (Cai et al. 2005; Afaf et al. 2016). However, conventional PCR gives simple identification of organism based on amplification of genetic material or DNA at the end point and is not quantitative; besides it has poor resolution as it can detect only at higher folds (10 or more) (Duarte et al. 2015). PCR has been used both for individual samples and bulk milk samples (Syring et al. 2012), or both (Baird et al. 1999). Threshold values for the number of cycles are considered as a diagnostic tool for the interpretation of PCR results for individual samples, particularly when the bulk sample interpretation seems to be difficult. PCR is effective for both subclinical (Moatamedi et al. 2007) and clinical mastitis (Syring et al. 2012).

In order to detect as well as quantify pathogens in relation to mastitis in milk, development of reverse transcription (RT)-PCR assays (Luminex, Biacore, Taqman, Lightcycler) have been accomplished. Use of these molecular methods has gained popularity for the differentiation of strains of bacteria within a species (Shome et al. 2011; El-Sayed et al. 2017). Such differences may be found in association with epidemiological differences as well as differences in relation to virulence and rate of cure and thus provide valuable information (Lakshmi 2016). RT-PCR provides identification at instant point of time and can be quantitative (qRT-PCR) and can detect even at smaller folds (2-folds) also (Keane et al. 2013). In cattle, a mastitis pathogen like *S. aureus* genotype B (GTB) can be isolated frequently and treatment against such kind of mastitis pathogen is not sufficient enough. An assay based on real-time PCR has been developed for the detection of this pathogen in bulk tank milk (BTM). Behera et al. (2018) developed a RT-PCR assay for detection of *Mycoplasma bovis* mastitis. It has also been used for the diagnosis of acute clinical mastitis caused by *Streptococcus aureus*, *S. uberis*, *S. dysgalactiae*, *Corynebacterium bovis* and *E. coli* in Finnish dairy cows in Finland during 2010–2012 (Vakkamäki et al. 2017). The pathogens most frequently encountered in case of mastitis (altogether 12 mastitis causing pathogens) are targeted by the assay. The throughput time of the assay is short for samples that are collected either fresh or are preserved. The advantage is that the assay is capable of detecting bacteria that are either dead or whose growth is inhibited, thereby decreasing false negative results. At routine recordings of milk promotion of the assay has been applied as an appropriate tool for detection of organisms causing mastitis from composite samples of milk. Implementation of the assay has been done in various European countries. It is mandatory to obtain Cycle threshold (Ct) values for the targets (bacterial DNA) for the purpose of scoring. There is involvement of 40 cycles in the thermal cycling protocol of the assay for the bacterial DNA target (Mahmmod 2013).

It must be kept in mind that at the initial stage a single pathogen had been targeted by mastitis tests based on PCR (Riffon et al. 2001). Subsequently, for detecting multiple pathogens at the same time in mastitic milk samples, multiplex PCR (m-PCR) also came into existence (Phuektes et al. 2001; Sarvesha et al. 2017; Hoque et al. 2018). The test is rapid and cost effective. The sensitivity of m-PCR is however low due to the competition between various primer sets for the DNA polymerase and deoxyribonucleotide triphosphate (dNTPs) (Phuektes et al. 2001; Gillespie and Oliver 2005; Amin et al. 2011). A m-PCR based on the 16S-23S rRNA spacer region has been developed for detecting *S. aureus*, *S. agalactiae*, *S. dysgalactiae* and *S. uberis* (Riffon et al. 2001; Phuektes et al. 2001; Shome et al. 2011). A multiplex PCR is available for detecting main pathogens of mastitis from buffalo milk also (Charaya et al. 2015). Using reaction-PCR, Preethirani et al. (2015) revealed that coagulase-negative staphylococci (NAS) were the most predominant bacteria (64.8%), followed by streptococci (18.1%), *E. coli* (9.8%) and *S. aureus* (7.3%) in causing mastitis in buffalo in South India. Another limitation of m-PCR is the problem in identifying solution phase m-PCR amplicons that may require other technologies, e.g. nucleotide sequencing (Edwards and Gibbs 1997).

In 2008, commercialization of PCR kit for mastitis was launched for the first time (Pathoproof, Thermo Fischer Scientific, Ltd.). Other kits such as Mastitis 4, DNA diagnostic (Risskov, Denmark) had also been made available. RT-PCR has been used in these commercial kits enabling quantification of DNA of bacteria. The results are automated, and with comparison to standard PCRs, throughput time is shorter (Koskinen et al. 2008). Evaluation of the Pathoproof assay has also been done in order to detect various pathogens causing mastitis under field conditions. The samples used for this purpose are milk collected from animals suffering from clinical or subclinical forms of mastitis along with spiked samples. The assay is proven to be highly accurate (95%), sensitive (100%) and specific (99–100%) at udder quarter and dairy animal levels (Koskinen et al. 2009; Mahmmod 2013).

Studies contributed that accurate diagnosis of bacterial pathogens involved in mastitis can be detected using another novel assay through high-resolution melt analysis (HRMA) of 16S rRNA sequences of the pathogens. In an experiment conducted for validation of this technique, RT-PCR was performed, sequencing was done, and the results revealed that HRMA can be used along with RT-PCR for confirmatory diagnosis of mastitic pathogens in conjugation with other laboratory culture techniques (Ajitkumar et al. 2012). Comparison of the results has been performed further with conventional bacteriological cultures. The sensitivity of PCR has been found to be much higher for detecting *S. aureus* and *S. uberis*; however, for *S. agalactiae* and *S. dysgalactiae*, significant differences have not been observed between microbiological culture and PCR. Further regarding these microbes, the m-PCR has been found to be less sensitive in comparison to the individual PCR (Phuektes et al. 2001). Simplex PCR has been able to detect as little as 5–50 pg of DNA of mastitis pathogens whereas multiplex PCR detected 50–500 pg. It is interesting to note that on the basis of 16S rRNA alone, m-PCR has been developed for simultaneous detection of nine bacterial pathogens of bovine mastitis and one single reaction can detect nine important bacteria, such as *S. agalactiae*, *S. dysgalactiae*, *S. uberis*, *S. epidermidis*, *S. aureus*, *S. haemolyticus*, *S. chromogenes*, *E. coli* and *Mycobacterium bovis*, directly from the milk samples. In comparison to traditional laboratory bacterial culture methods and 16S rRNA sequencing, this assay is 88% sensitive and 98% specific. This molecular assay is helpful in monitoring the epidemiological status of mastitis producing bacteria and assessing the microbiological quality of milk, since it has been sensitive enough to detect the small quantity of 50 pg of bacterial DNA (Ashraf et al. 2017).

A PCR assay has been developed for diagnosing *S. agalactiae* directly in bovine milk on the basis of the 16S rRNA gene. The study is indicative of the fact that the PCR is highly specific, sensitive and rapid; representing an innovative tool for detecting *S. agalactiae* in milk samples (Martinez et al. 2001). Another PCR assay has been developed for identification of *S. agalactiae*, *S. dysgalactiae*, and *S. uberis* in case of bovine IMI. The assay was found to be rapid, sensitive as well as specific. On the basis of 16S rRNA gene (in case of *S. agalactiae* and *S. dysgalactiae*) and 23S rRNA gene (in case of *S. uberis*), specific primers were designed. Species of bacteria that are closely related phylogenetically can be discriminated by using such primers. The detection limit has been found to be 3.12 × 10^2^ CFU ml^−1^ (with the step of pre-PCR lyses enzymatically) and 5 × 10^3^ CFU ml^−1^ (without the step of pre-PCR lyses enzymatically). The researchers have observed that implementation of such PCR assays can be done promptly in diagnostic microbiological laboratories and thereby can greatly aid to the prevention of mastitis in cattle (Riffon et al. 2001).

It may happen sometimes that a sample may be found negative upon examination by bacteriological culture but may be found positive by PCR. This is mainly because the sample may have very less concentration of pathogens, the media and/or culturing conditions are not favorable, or the milk sample may contain residual antibiotics (Phuektes et al. 2001; Moatamedi et al. 2007). Nevertheless, while using PCR technologies for the detection of pathogen from mastitic milk samples, researchers should keep in mind that there are chances to obtain results that are false positive in nature. This may be due mixing of normal and affected milk from healthy and mastitic cows. Moreover, differentiation between non-viable as well as viable bacteria is not possible by using PCR. Because of all these reasons, it is suggested to the dairy advisors to take the help of information available, viz., history of the mastitic condition, udder examination (clinically), history of therapeutic regimen followed previously, SCC along with the PCR results to take correct decision (Mahmmod 2013).

#### 2.2.3. Sequencing/molecular typing methods

Sequencing has become an important mastitis diagnostic tool, not only for the species/subspecies/strain level identification of microorganisms, but also overcoming the resolution problems. Differentiation of various bacterial strains within a species can be done by the use of these molecular techniques. Such differences are crucial as the various strains may be associated with epidemiological differences and differences in virulence along with rates of cure. For this purpose, genotyping/fingerprinting has been found to be a suitable approach. Such methods are in frequent use in case of research related to epidemiological studies (Zadoks and Schukken 2006). Various molecular methods, such as ribotyping (Choudhary et al. 2018), pulsed-field gel electrophoresis (PFGE) (Santos-Sanches et al. 2015; Pumipuntu et al. 2019), amplified fragment length polymorphism (AFLP) (Sharma et al. 2006; Mohajeri et al. 2016), random amplified polymorphic DNA (RAPD) (Tomazi et al. 2018), and multilocus sequence typing (MLST) (Shibata et al. 2014; Rosales et al. 2015; Pumipuntu et al. 2019) have been frequently employed for the purpose of genotyping. DNA is used in these methods following digestion of the DNA with restriction enzymes, after employing PCR to amplify the DNA, after analyzing the sequence of DNA or by combining all these approaches (Zadoks and Schukken 2006; Lam et al. 2009; Choudhary et al. 2018; Rainard et al. 2018). At strain level, multi-locus variable number tandem repeat analysis (MLVA) and at species level, analysis of transfer DNA intergenic spacer length polymorphism as well as ribotyping can be performed (Carretto et al. 2005; Pinho et al. 2012).

PFGE along with binary interspace (IS) typing can be applied with success for the purpose of genetic analysis of *S. aureus* isolates from bovine mammary secretions. Binary IS typing is found to be a robust method that can be used with simplicity and holds great promise of becoming a powerful tool for characterizing strains of the bacteria (Zadoks et al. 2000). Large molecules of DNA are separated by PFGE wherein electric field is used. When compared to agarose gel electrophoresis, PFGE helps in getting better size resolution. The technique is often employed for tracking of pathogens and is proven to be a typing scheme of great value for detection and differentiation of bacterial strains. The protocol of PFGE has been found to be highly suitable for the purpose of study of streptococci responsible for causing bovine mastitis (Santos-Sanches et al. 2015). PFGE has been found to be helpful for the purpose of basic epidemiological investigation for detection of ovine mastitis causing strains of *S. aureus*. The most suitable discriminatory technique to distinguish strains of *S. aureus* is PFGE. It is important to mention in this regard that the coagulase (*coa*) as well as protein A (*spa*) types can be correlated with PFGE types (Ciftci et al. 2009). Antimicrobial resistance profile of *S. aureus*, isolated from cases of bovine mastitis, is recorded in various parts of globe including China and is helpful in studying the epidemiological pattern of resistant bacterial strains. Vast diversity is observed among strains of causative bacteria resulting into modified epidemiological pattern of infection within dairy animals due to genetic variation (Shi et al. 2010). Both PFGE as well as MLST have been employed for the purpose of genotyping for characterization of various strains of *S. aureus* causing bovine mastitis (Pumipuntu et al. 2019). This is followed by the submission of the strains to a characterization scheme. This scheme consists of wide variety of assays in relation to pathogenicity and resistance to antibiotics (Delgado et al. 2011; Pumipuntu et al. 2019). New strains of *S. uberis* responsible for recurrent infection has been identified by the use of PFGE (Abureema et al. 2014).

It is also interesting to note that by the use of PFGE along with MLST and ribotyping the genetic relationship between isolates of group B streptococci in human and bovine mastitis can be detected (Oliveira et al. 2006). Multiplex-PCR was conducted by using specific primers designed to help detect either group on the basis of the sequences of DNA. The results were indicative of the fact that several isolates of *S. aureus* (from clinical as well as subclinical cases) along with microbes in bulk milk can be detected by using such primers; thereby facilitating early diagnosis of infection in dairy animals caused by *S. aureus*. MLST has been developed for differentiation of isolates of *S. uberis* from milk. Two new clonal complexes, viz., sequence type (ST-86) along with ST-143 have been identified by the application of this particular technique (Pullinger et al. 2006; Tomita et al. 2008). MLST has been employed along with m-PCR for capsular typing (genotypic) and detection of virulence gene (eight different sequence types, viz., ST-61, ST-67, AT-91, ST-103, ST-146, ST-226, ST-314, and ST-570 have been identified for the purpose of addressing the molecular epidemiology of several isolates of *S. agalactiae*). Clustering of these STs have been done in five clonal complexes (CCs), viz., CC17, CC64, CC67, CC103 and CC314 along with a singleton, ST-91 (Carvalho-Castro et al. 2017).

In case of resistance to mastitis for determination of genome-wide markers (linked to quantitative trait locus), AFLP technique has been used on (selective) DNA pools (Sharma et al. 2006).

Gonçalves et al. (2010) attempted to develop a molecular approach for detecting two RAPD groups of *S. aureus* with rapidity along with accuracy and for this purpose characterization of several isolates of the organism from infected animals was done by RAPD followed by cloning and sequencing of the genome fragments.

For quick screening of suspected milk samples for clinical mastitis, LAMP has come up as an effective, fast and novel molecular technique. By the use of four specific primers designed as per various sequences (six in particular) of the *nuc* gene, LAMP has been developed to detect *S. aureus* rapidly in mastitic dairy cows. Additionally, verification of the sensitivity as well as specificity of the assay was done along with comparison with PCR. Sensitivity of LAMP was noted to be 100 times higher than PCR as it detected minimal concentration of 1 × 10^2^ CFU ml^−1^, whereas PCR detected 1 × 10^4^ CFU ml^−1^. LAMP was more specific for detection of *Staphylococcus aureus* than other strains including *E. coli*, *S. agalactiae*, *S. typhimurium*, and *S. epidermidis*. It is interesting to note that completion of such assay can be done at 62.5 °C very quickly (i.e. 45 min) (Tie et al. 2012). Again, for facilitating management of *S. agalactiae* on farm, LAMP has been developed to quickly screen milk samples. It has been documented that 108 strains of *S. agalactiae* were confirmed by laboratory culture techniques, LAMP and PCR, as cause of bovine mastitis (Bosward et al. 2016). Kawai et al. (2017) selected pathogenic microorganisms belonging to 12 genera causing mastitis, optimized the DNA amplification conditions by LAMP using 32 primers and DNA chip which could measure all pathogens at the same time. This method allows highly sensitive and fast detection of etiological pathogens of mastitis. Total positive concordance rate was 85.0% and that of negative concordance was 86.9% against the set positive concordance of 100% for culture. Ashraf et al. (2018) suggested that the LAMP targeting uvrC and gyrB genes could be a rapid and accurate tool for diagnosis *M. bovis* mastitis.

To promote rapid identification of milk pathogens responsible for clinical mastitis in dairy cows, two diagnostic approaches were used altogether in one system known as Accumast and its accuracy, sensitivity and specificity was tested on farm level. It involved traditional bacterial culture method on farm and isolate sequencing based upon 16S rRNA. Among bacterial isolates, *Staphylococcus* spp., *Streptococcus* spp., *E. coli* and *Enterococcus* spp. were found. The sensitivity, specificity and accuracy of Accumast were 82.3, 89.9 and 84.9%, respectively (Ganda et al. 2016).

Researchers analyzed the DNA-based microbial diversity of bovine mastitic milk by using metagenomic pyrosequencing of bacterial 16S rRNA genes from 136 mastitic milk and 20 disease-free milk samples. They documented *Trueperella pyogenes*, *S. dysgalactiae* and *S. aureus* as first, second and third most prevalent strains, respectively, while *E. coli*, *Klebsiella* spp. and *S. uberis* were present in few diseased mastitic animals (Oikonomou et al. 2012). Researchers have also worked in developing a fast and sensitive immunosensor assay for measuring an important acute phase protein haptoglobin (Hp) secreted during udder inflammation and particularly useful for detecting cases of subclinical mastitis. Results based upon 20 milk samples without showing clinical signs and with SCC of more than 5 × 10^5^ cells ml^−1^ were promising and recommended the use of this biosensor in dairies under field conditions for quick diagnosis (Tan et al. 2012).

Five dairy farms in Saudi Arabia were screened using two methods of MALDI biotyper (MBT) and Vitek(™) 2 compact system for prompt identification of *Staphylococcus* species isolated from bovine mastitis suspected for staphylococcal involvement and results revealed that it detected 198 isolates of *Staphylococcus* species and hence MBT is a useful potential diagnostic candidate for quick diagnosis (Elbehiry et al. 2016). In this regard, it is important to note that the development of MLST has further aided to these diagnostics (Shibata et al. 2014).

#### 2.2.4. Advanced specific mastitis diagnostics

MALDI-TOF (Schabauer et al. 2014; Cameron et al. 2017), ultra-performance liquid chromatography-quadrupole-time of flight mass spectrometry (Xi et al. 2017), and specific immunoassays (Addis et al. 2016; Hussein et al. 2018) are the latest and novel diagnostic methods for mastitis.

MALDI-TOF MS which is based on the principle of mass spectroscopy can be used for determination of the species of bacteria as well as strains or their proteins in a very short time (within minutes) (Elbehiry et al. 2016; Magro et al. 2018). Among popular proteomic approaches MALDI-TOF was used for recognition and mapping of surface associated proteins of *S. aureus* isolates recovered from bovine mastitis. Protein extracts of *S. aureus* obtained from bovine mastitis cases was treated with lysostaphin and used to prepare a reference map of surface proteins by 2D-GE technique to know about the specific characteristics of pathogenic bacteria for diagnosis (Taverna et al. 2007).

Using MALDI-TOF, Schabauer et al. (2014) identified bovine mastitis associated Gram-positive, catalase-negative cocci. Similarly, Cameron et al. (2017) identified bovine-associated NAS. The reliability of the technique is high and it can be used easily and also is very cost effective. This technique can be used for potentially replacing and/or complementing identification by phenotypic methods (conventionally) as it is 100% specific and sensitive. MALDI-TOF MS is applicable only to specific spectra databases of the existing protein profiles of bacteria for the purpose of identification. It is also important to note that the technology is still found to be not so much economical for use in diagnostic laboratories widely (Bizzini and Greub 2010; Raemy et al. 2013).

MALDI-TOF can be used both on culture and non-culture, milk or non-milk samples (Singhal et al. 2015; Barreiro et al. 2017; Mahmmod et al. 2018); however, utilization of MALDI-TOF directly on milk seems to be less reliable unless bacterial concentration is high (Barreiro et al. 2017; Klaas and Zadoks 2018). Though for identification of bacterial species and strains, it has 100% specificity and sensitivity but for identification bacterial protein profile spectra is limited (Bizzini and Greub 2010; Duarte et al. 2015).

MALDI-TOF spectrometric analysis is used for modern phenotypic testing which is based on proteomics (Schabauer et al. 2014; Cameron et al. 2017). It can directly be applied to milk samples (Barreiro et al. 2017).

Ultra-performance liquid chromatography-quadrupole-time of flight mass spectrometry has been evaluated for detection of milk metabolomics in dairy cows with subclinical or clinical mastitis and has been found quite effective (Xi et al. 2017). These technologies are costly and usually not affordable for routine mastitis diagnostic purposes (Duarte et al. 2015). Some novel immunoassays are being developed for estimation of biomarkers of mastitis (amyloid A, milk, blood or serum biochemical) in dairy animals (Qayyum et al. 2016; Hussein et al. 2018). These are comparatively convenient and do not require costly infrastructure; however for specificity, specific immunoassays are required based on species-specific antibodies.

#### 2.2.5. Specific immunoassays

Immunoassays could pave way for efficient diagnosis of clinical or subclinical mastitis (Bu et al. 2015; Jaeger et al. 2017). Jaeger et al. (2017) utilized ELISA for milk amyloid A as promising biomarker for detection of subclinical mastitis. Bu et al. (2015) explored indirect ELISA for Sip protein of *S. agalactiae* for diagnosis of bovine mastitis. Enzyme-linked immunosorbent assays have been developed for only few selected pathogens, *viz*., *S. aureus*, *Listeria monocytogenes*, and *E. coli*. *S. aureus* antibody test kit (SAATK) has been developed for primary screening of cows for *S. aureus* mastitis (Fox and Adams 2000; Viguier et al. 2009). The advantage of magnetic bead-based ELISA over conventional ELISA is its rapidness for diagnosis of *S. aureus*; reagents required are comparatively less along with lesser manipulation. Antibodies against *S. aureus* can also be detected by employing flow cytometry, which in comparison to bacteriological tests, helps in generating results in an early stage (Viguier et al. 2009). Researchers have developed and standardized ELISA for detecting and assessing cathelicidin proteins present in milk of mastitic ewes. Cathelicidin are antibacterial small proteins related to innate immunity secreted in the milk in case of mastitis. Study was performed using 705 milk samples from sheep farms and findings revealed the presence of cathelicidins along with significant SCC and bacterial culture confirms the ovine mastitis (Addis et al. 2016). ELISA may help in diagnosis of mastitis through novel biomarkers (Hussein et al. 2018). The disadvantages of these methods include non-specificity due to cross-reactions, hence less accuracy, high cost, and requirement of infrastructure and technically skilled persons (Garcia-Cordero et al. 2010; Duarte et al. 2015, 2017).

#### 2.2.6. Mastitis specific biomarkers

There are many milk or serum based indicative biomarkers that can be estimated for the mastitis diagnosis. Alteration in their levels can be significantly correlated with mastitis. There is release of various enzymes in the milk due to the immune responses of the animals against various infectious conditions and alteration in the permeability (increased) of the blood vasculature. There is a tendency of reduction of the enzymes that deal with synthesis of milk along with increased activity of the enzymes found in relation to inflammation (Pyörälä 2003). There is exponential increase in the activities of enzymes that originate from phagocytes such as *N*-acetyl-d-glucosaminidase (NAGase), milk LDH, ALP, arginase and catalase along with β-glucoronidase (Oliszewski et al. 2004; Kandemir et al. 2013; Preethirani et al. 2015). Significant increase in milk LDH activity, ALP activity and phosphorus levels and decrease in calcium levels were noted in mastitis-affected cows (Afaf et al. 2016). There is also an increase in the enzymatic activities of plasminogen in blood (activated totally to plasmin) and a proteolytic enzyme that is responsible for the degradation of fibrin along with casein (Pyörälä 2003). For the diagnosis of mastitis, various acute phase proteins can be successfully used as biomarkers, *viz*., serum amyloid A (SAA) (Hussein et al. 2018) and Hp (Kalmus et al. 2013) as well as enzymes like NAGase (Kalmus et al. 2013), LDH, and ALP (Persson et al. 2014) and an enzyme in the cell cytoplasm (Hiss et al. 2007). It has been revealed that in clinically healthy cows LDH shows least variation between milking in comparison to haptoglobin, SAA and NAGase. There is an increase in the activity of enzymes, e.g. LDH and NAGase in milk and these changes can be measured by colorimetric as well as fluorometric assays at early stage of mastitis (Larsen 2005; Duarte et al. 2015). During mastitis, release of proteins in milk may be due to proteolysis caused by either bacteria or endogenous proteases. Peptide biomarkers can be used for diagnosing mastitis, which also helps in discrimination between microbial (bacterial) causes of mastitis. A panel of biomarkers comprising of several peptides has been revealed by the use of liquid chromatography; capillary electrophoresis as well as mass spectrometry with high sensitivity and even higher specificity (Mansor et al. 2013).

Though these biomarkers are vulnerable to change in other disease conditions also hence they serve as general type of biomarkers as discussed previously however when alterations are correlated to mastitis they can specify the diagnosis, and especially when the milk or udder based alterations are described they can be mastitis specific biomarkers. Acute phase proteins (APPs) including haptoglobin (Hp), CRP and mammary associated serum amyloid A3 (M-SAA3) are also used as biomarkers for diagnosing bovine mastitis. These proteins actually increase in milk during the process of inflammation but are found in much lower concentrations in milk samples that are healthy (Gronlund et al. 2003; Akerstedt et al. 2007). Further these acute phase proteins vary with reference to causative agent and type of mastitis (Thomas et al. 2018). *E. coli*, *S. uberis* and *S. dysgalactiae* mastitis results in higher increase in milk APP levels as compared to other pathogens (Thomas et al. 2018). CRP and Hp levels vary between clinical and subclinical mastitis however M-SAA3 shows non-significant change (Thomas et al. 2018). Diagnostics based on these biomarkers can be helpful in differentiating healthy cows and the ones affected with clinical or subclinical mastitis. Use of immunoassays is indeed frequent for the detection of acute phase proteins for diagnosing bovine mastitis like the development of ELISA’s aimed at Hp in milk with a limit of detection of 0.07 μg ml^−1^, SAA and NAGase (Szczubial et al. 2012). Discovery of novel mediators of inflammation is concerned. ELISAs have much less role due to the availability of restricted numbers of antibodies that are bovine isotype-specific in nature (Duarte et al. 2015). Research has been conducted for improving the strategies for diagnosis of bovine mastitis associated with *E. coli* by combining meta-analysis as well as machine learning (data mining tools) which have the capability of detecting genes that are most informative. Such genes can act as biomarkers for *E. coli*-induced mastitis in cattle and include *ZC3H12A*, *CXCL2*, *GRO*, and *CFB* (Sharifi et al. 2018). Solexa (Illumina) sequencing (sequencing method based on reversible dye-terminators that enable the identification of single bases as they are introduced into DNA strands) along with bioinformatic tools have been employed for analysis of miRNA in case of experimentally induced mastitis (caused by *S. aureus*) indicating miRNAs to be potential biomarkers for diagnosing bovine mastitis (Li et al. 2015). Bochniarz et al. (2018) reported that serum and milk concentrations of tryptophan, kynurenine and kynurenic acid were lower in cows having subclinical mastitis caused by non-*aureus* staphylococci. These findings may be used for diagnosis of bovine mastitis as this reduction might be a marker indicating mastitis too.

## 3. Conclusion and future perspectives

Improving of mastitis diagnostics helps in early, rapid and accurate diagnosis of (subclinical) mastitis. This also minimizes economic losses and safeguards public health through prevention of mastitis and better management of dairy animals. Advancements in technologies have led to dramatic shift from application of conventional diagnosis of less specificity and/or sensitivity to highly sophisticated, rapid and reliable molecular diagnosis of high accuracy. However, despite success in advanced diagnostic tests, conventional tests aid in the confirmation of diagnosis when used in combination, and are helpful in preliminary screening when used alone. Despite this, advanced molecular diagnostics have immense capability of transforming diagnostic and management aspects of mastitis and have found place in routine laboratory protocols and in future with simplification of procedures can become boon for theranostics.

Several diagnostic tests are in use on routine basis for diagnosis of mastitis and include CMT, bacterial culture, SCC along with PCR as most commonly used tools to serve the purpose. In cows with clinical mastitis sensitivity of CMT in probability of predicting infection (36–91%) is higher than in cows without clinical mastitis (5–68%). Sensitivity of PCR (∼91%) is higher than that of CMT (∼61%) which is higher than that of bacterial culture (∼53%). However, specificity of PCR is higher (∼99%) than bacterial culture (∼89%) which is higher than that of CMT (∼65%). On comparative basis, SCC has 28–98% sensitivity and 4–89% specificity while as CMT has 4–66% sensitivity and 54–97% specificity in identifying infected quarters in early lactation. Thus PCR shows better diagnostic performance than the conventional diagnostic tests (BC and CMT) hence can be used for accurate diagnosis. For getting extensively robust results, PCR and bacteriological culture are used together. But this approach is not feasible to get a therapeutic decision. Advances in the knowledge of proteomics and genomics have led to the discovery of several biomarkers; the feasibility of proteomic research for reliable biomarkers to detect mastitis at an early stage as well as to determine the efficacy of drugs is high and quite noteworthy. Assays developed on the basis of the knowledge of proteomics and genomics are highly sensitive in nature. This in turn provides quantitative information in addition on the inflammation level (both on-line as well as on-site). Biochemical substances act as indicators of mastitis, acute phase proteins including haptoglobin, CRP, mammary associated serum amyloid A3 reflect gravity of change and expression of genes or proteins reflect response to mastitis at molecular level, all serving as diagnostic biomarkers. Nucleotide sequencing, mass spectrometric analysis or specific immunoassays are the novel approaches for diagnosing the mastitis. Conventional tests being subjective in nature will help in preliminary identifying the etiological agent or the mastitis-associated changes, while the quantitative and confirmatory aspects of the novel tests enable the confirmation of etiology and quantification of the amount of changes. It is interesting to note that these assays are rapid, specific, sensitive and economic. Further, automated monitoring systems can be incorporated with advanced technologies due to advances in the field of microfluidics in recent times and it will help to detect mastitis with greater sensitivity as well as rapidity. However, both specializations in training along with experience for interpretation of results are needed for implementing the advanced technologies for efficient mastitis diagnosis and improving udder health management.
